# Rural-urban differentials of prevalence and lifestyle determinants of pre-diabetes and diabetes among the elderly in southwest China

**DOI:** 10.1186/s12889-023-15527-9

**Published:** 2023-03-30

**Authors:** Yi Zhao, Hui-fang Li, Xia Wu, Guo-hui Li, Allison Rabkin Golden, Le Cai

**Affiliations:** 1 School of Public Health, Kunming Medical University, 1168 Yu Hua Street Chun Rong Road,Cheng Gong New City, Kunming, 650500 China; 2 The First Affiliated Hospital of Kunming Medical University, Kunming, 650032 China; 3 The Second Affiliated Hospital of Kunming Medical University, Kunming, 650032 China

**Keywords:** Diabetes, Determinants, Rural-urban differentials, China

## Abstract

**Background:**

Diabetes has become a major public health problem in China. A better understanding of diabetes determinants and urban-rural differences is essential to crafting targeted diabetes prevention measures for the elderly living in both urban and rural areas. This study aimed to compare rural-urban differentials in prevalence and lifestyle determinants of pre-diabetes and diabetes among the elderly in southwest China.

**Methods:**

A cross-sectional health interview and examination survey was conducted among individuals aged ≥ 60 years in both a rural and urban area of China. Anthropometric measurements, including height, weight, and waist circumference, as well as blood pressure and fasting blood glucose measurements were taken. Associated risk factors for pre-diabetes and diabetes were evaluated using multivariate logistic regression analysis.

**Results:**

In total, 1,624 urban residents and 1,601 rural residents consented to participate in the study. The urban prevalence of pre-diabetes and diabetes (46.8% and 24.7%, respectively), was higher than the rural prevalence (23.4% and 11.0%, respectively, *P*<0.01). Urban elderly participants had markedly higher prevalence of obesity, central obesity, and physical inactivity than their rural counterparts (15.3%, 76.0%, and 9.2% vs. 4.6%, 45.6%, and 6.1%, *P*<0.01). In contrast, rural elderly adults had higher prevalence of smoking than urban ones (23.2% vs. 17.2%, P<0.01). Obese (OR 1.71, 95% CI 1.27–2.30 vs. OR 1.73, 95% CI 1.30–3.28) and centrally obese participants (OR 1.59, 95% CI 1.18–2.15 vs. OR 1.83, 95% CI 1.32–2.54) were more likely to suffer from diabetes in both urban and rural regions. Furthermore, urban current smokers had a higher probability of suffering from diabetes (OR 1.58, 95% CI 1.11–2.25), while hypertension was positively associated with the prevalence of diabetes in the rural area (OR 2.13, 95% CI 1.54–2.95). Obese participants in the rural area were more likely to suffer from pre-diabetes (OR 2.50, 95% CI 1.53–4.08), while physical inactivity was positively associated with prevalence of pre-diabetes in the urban area (OR 1.95, 95% CI 1.37–2.80).

**Conclusion:**

Pre-diabetes and diabetes are more prevalent among urban older adults than their rural counterparts in southwest China. The identified rural-urban differentials of lifestyle factors have significant impacts on prevalence of pre-diabetes and diabetes. Thus, tailored lifestyle interventions are needed to improve diabetes prevention and management among the elderly in southwest China.

## Background

Diabetes is a growing public health challenge globally, with type 2 diabetes contributing up to 90% of all diabetes cases [1]. The International Diabetes Federation estimates the global diabetes prevalence in 2021 was 10.5% (536.6 million people), and will rise to 12.2% (783.2 million people) in 2045 [2]. The prevalence of pre-diabetes, the preceding condition of diabetes defined as blood glucose levels that are higher than normal, but lower than diabetes thresholds [3], is also rising worldwide [4]. As those with pre-diabetes are at a high risk for developing diabetes, the rapid increase in the prevalence of diabetes and pre-diabetes is translating into a growing diabetes prevention and treatment challenge.

Over the last several decades China’s population has steadily aged. In China’s most recent census, 260 million people were 60 years and older, accounting for 18.7% of the total population [5]. Older adults are at higher risk for diabetes and the prevalence of diabetes increases with age [6, 7]. Over the past 30 years, the prevalence of diabetes in China has increased 17-fold, from 0.67 to 11.6% due to socioeconomic development, the aging of the population, lifestyle changes, obesity, and increasing urbanization [8, 9]. Diabetes is more prevalent in the urban Chinese population versus rural population [7, 10]. At the same time, the prevalence of pre-diabetes among the elderly aged over 60 years has also increased significantly in China, rising from 24.5% in 2008 [7] to 45.8% in 2013 [11].

A better understanding of diabetes determinants and urban-rural differences is essential to crafting targeted diabetes prevention measures for the elderly living in both urban and rural areas. Previous research established several factors associated with increased risk of diabetes, including aging [6, 7, 12], obesity [7, 12, 13], living environment [6, 7], physical inactivity [13, 14], hypertension [13, 15] and socioeconomic status [16]. However, there is limited research on the rural-urban differentials of the prevalence and determinants of diabetes in both China [17] and other countries [18, 19]. Moreover, few studies have focused on health disparities between the urban and rural elderly populations. Due to uneven economic development, education levels, and living environments across urban and rural regions in China, health disparities between the urban and rural elderly are rapidly growing [20]. Thus, this study aimed to address this growing challenge by uncovering the rural-urban differentials in prevalence and determinants of pre-diabetes and diabetes among the elderly in southwest China.

## Methods

### Data sources and study population

Yunnan Province, one of China’s poorest provinces, is located in southwest China, bordering Myanmar, Laos, and Vietnam. It has 129 counties and a total population of 47.2 million people (as of 2020). Kunming is the capital of Yunnan, and has 7 urban districts, 7 counties, and a recorded population of 8.46 million (as of 2020). The present study employed a cross-sectional health interview and examination survey conducted from July 2019 to October 2019 in one rural area and one urban area of Kunming. The rural and urban areas were selected using a four-stage, stratified, random sampling method. In the first stage of selection, Kunming was divided into two strata: rural regions and urban regions, with one area then randomly chosen from each stratum. In the second stage, each selected area was then further classified into three categories according to wealth distribution (per capita GDP): low, medium, and high. From each of these three categories, one street or one township was then randomly selected, for a total of 6 streets and 6 townships. In the third stage, three communities and three villages were randomly selected from the selected streets and townships using probability proportion to size sampling method (PPS sampling). In the fourth and final stage, eligible participants aged 60 years and over were selected to be invited to participate within each chosen community or village, using simple random sampling. Inclusion criteria were (1) participants aged ≥ 60 years; and (2) participants residing in the selected community or village ≥ 5 years and willing to participate in the study. Exclusion criteria were (1) participants aged < 60 years; (2) those with cognitive dysfunction or inability to communicate with the interviewers; and (3) those refused to provide informed consent to participate in the study.

## Data collection and measurement

Kunming Medical University medical students were selected and trained as interviewers for data collection. These trained interviewers administered a pre-tested and structured questionnaire via a face-to-face interview to collect data on demographic characteristics and lifestyle factors for all participants. Anthropometric measurements, fasting blood glucose (FBG), and blood pressure (BP) tests were also collected and recorded.

Physicians at the First Affiliated Hospital of Kunming Medical University performed the FBG tests. FBG was measured the morning after participants completed a minimum of 8 h of overnight fasting. Participants who reported that they had not fasted at least 8 h were invited to receive an additional FBG test on another day.

Using a mercury sphygmomanometer, BP was measured three times for all participants, in accordance with American Heart Association recommendations [21]. After participants rested for five minutes in a seated position, the three readings were averaged and the final averaged BP value was recorded.

Anthropometric measurements, including weight, height, and waist circumference, were measured using standardized equipment and procedures as described in the World Health Organization (WHO) STEPS manual [22]. Body mass index (BMI) was also recorded and calculated as weight (kg) divided by squared height (m^2^).

### Ethical approval

The Ethics Committee of Kunming Medical University approved this study prior to the commencement of the research.

### Definitions

Diabetes was defined as FBG ≥ 7.0 mmol/l (126 mg/dl), a reported use of antidiabetic medications within the two weeks prior to the study, or a reported previous diagnosis of diabetes by a healthcare professional. Pre-diabetes was defined as FBG ≥ 6.1 mmol/l and < 7.0 mmol/l.

Hypertension was defined as an average systolic blood pressure ≥ 140 mmHg, and/or an average diastolic blood pressure ≥ 90 mmHg, or self-reported use of an antihypertensive drug in the past 2 weeks [23]. A previous diagnosis of hypertension by a health professional was also considered as hypertension in this study.

Obesity was defined as a BMI of 28.0 kg/m^2^ or higher [24]. Central obesity was defined as waist circumference at or above 90 cm in men, and at or above 80 cm in women, based on WHO recommendations for Asian adults [24].

Current smoker was defined as having smoked more than 100 cigarettes or at least 150 g of tobacco in one’s lifetime, and smoked any form of tobacco product on a daily basis at the time of the survey. Current drinker was defined as drinking alcohol regularly on 12 days or more during the 12 months preceding the study. Physical inactivity was defined as failure to do at least 150 min of moderate-intensity physical activity throughout the week, or failure to do at least 75 min of vigorous-intensity physical activity throughout the week [25].

### Statistical analysis

Continuous variables were expressed as mean ± standard deviation (X±S), while categorical variables were described as counts and percentages. A chi-squared test was used to compare categorical variables, while t-tests were used to analyze continuous measures. The overall prevalence of pre-diabetes and diabetes were adjusted for age and sex by direct standardization to the total population of the two study areas. Multivariate logistic regression analysis was used to test the association of prevalence of pre-diabetes and diabetes with other variables (age, sex, level of education, BMI, obesity, central obesity, hypertension, current drinkers, current smokers, and physical inactivity). Associations were expressed as odds ratios and 95% confidence intervals (CI). *P* values < 0.05 were considered significant. All statistical analyses were conducted using SPSS version 22.

## Results

The number of participants aged 60 years and over, invited to join in the survey was 1,700 in urban area, and1,700 in rural area. Of these, 1,624 urban residents and 1,601 rural residents consented to participate in the study, representing a response rate of 95.5% and 94.2%, respectively.

Table 1 displays the demographic characteristics of the study population. Proportion of each age group and gender among the participants did not differ between the urban and the rural regions (*P* > 0.05). However, urban participants had markedly higher levels of education as well as a higher prevalence of diagnosed hypertension by a health professional, obesity, central obesity, and physical inactivity than their rural counterparts (*P*<0.01).

**Table 1:** Demographic and lifestyle characteristics and mean values of BP, FBG, and anthropometric measurements among the study population

Characteristic	Rural area			Urban area		
	Male (n = 741)	Female (n = 860)	All (n = 1601)	Male (n = 752)	Female (n = 872)	All (n = 1624)
Age group (%)						
60–64 years	152(20.5)	208(24.2)	360(22.5)	151(20.1)	212(24.3)	363(22.4)
65–69 years	195(26.3)	209(24.3)	404(25.2)	208(27.7)	213(24.4)	421(25.9)
70–74 years	153(20.6)	201(23.4)	354(22.1)	154(20.5)	205(23.5)	359(22.1)
75–79 years	136(18.4)	145(16.9)	281(17.6)	137(18.2)	142(16.3)	279(17.2)
80–84 years	73(9.9)	69(8.0)	142(8.9)	69(9.2)	68(7.8)	137(8.4)
85–89 years	25(3.4)	25(2.9)	50(3.1)	25(3.3)	26(2.7)	51(3.1)
≥ 90 years	7(0.9)	3(0.3)	10(0.6)	8(1.1)	9(0.7)	14(0.9)
Level of education (%)						
Primary (grade 1–6) or lower	362(48.9)	597(69.4)*	959(59.9)	11(1.5)**	28(3.2)**	39(2.4)**
Middle (grade 7–9) or higher	379(51.1)	263(30.6)	642(40.1)	741(98.5)	844(96.8)	1585(97.6)
Current smokers (%)	365(49.3)	6(0.7)	371(23.2)	274(36.4)**	6(0.7)	280(17.2)**
Current drinkers (%)	239(32.3)	7(0.8)	246(15.4)	248(33.0)	4(0.5)	252(15.5)
Obesity (%)	28(3.8)	46(5.3)	74(4.6)	125(16.6)**	124(14.2)**	249(15.3)**
Central obesity (%)	261(35.2)	469(54.5)	730(45.6)	544(72.3)**	691(79.2)**	1235(76.0)**
Hypertension (%)	333(44.3)	331(38.0)	664(40.9)	299(40.4)	397(46.2)	696(43.5)
Diagnosed hypertension by a health professional (%)	203(27.4)	274 (31.9)	477 (29.8)	296 (39.4)	345(39.6)	641 (39.5)**
Physical inactivity (%)	44(5.9)	54(6.3)	98(6.1)	66(8.8)*	83(9.5)*	149(9.2)*
Height (cm, mean ± SD)	162.3 ± 7.3	151.5 ± 6.6	156.5 ± 8.8	164.9 ± 6.0*	153.6 ± 6.2*	158.8 ± 8.3*
Weight (kg, mean ± SD)	57.5 ± 9.8	51.1 ± 9.3	54.0 ± 10.0	68.4 ± 9.9**	58.5 ± 8.2**	63.0 ± 10.3**
Waist circumference (cm, mean ± SD)	81.3 ± 9.8	80.6 ± 9.8	80.9 ± 9.8	89.7 ± 8.1**	86.1 ± 8.3**	87.8 ± 8.4**
BMI (kg/m^2^, mean ± SD)	21.8 ± 3.5	22.2 ± 3.6	22.0 ± 3.6	25.1 ± 3.1**	24.8 ± 3.6**	25.0 ± 3.4**
Systolic BP (mm Hg, mean ± SD)	125.0 ± 19.3	126.2 ± 19.2	125.6 ± 19.3	136.4 ± 17.5	135.0 ± 16.3	135.7 ± 16.9
Diastolic BP (mm Hg, mean ± SD)	78.8 ± 11.8	79.5 ± 11.2	79.2 ± 11.5	82.6 ± 11.2	79.0 ± 9.8	80.7 ± 10.6
FBG (mmol/l, mean ± SD)	5.9 ± 1.5	6.2 ± 2.2	6.1 ± 1.9	6.7 ± 2.6**	6.7 ± 2.2**	6.7 ± 2.4**

* P < 0.05, ** P < 0.01

BMI = body mass index

BP = blood pressure

FBG = fasting blood glucose

SD = standard deviation

Table 2 presents the age-standardized prevalence of diabetes and pre-diabetes by geographic region. The age-standardized prevalence of pre-diabetes and diabetes in the urban older adult study population (46.8% and 24.7%) was significantly higher than in the rural population (23.4% and 11.0%), and these higher rates were also found among subgroups stratified by gender, age, and education ( *P*<0.01). In both the urban and rural area, the prevalence of pre-diabetes decreased as age increased, while the prevalence of diabetes increased with age only in the urban region.

**Table 2:** Age-standardized prevalence of pre-diabetes and diabetes by sex, age, and level of education among rural and urban elderly people of Yunnan Province, China

Characteristic	Pre-diabetes		Diabetes	
	Rural n (%)	Urban n (%)	Rural n (%)	Urban n (%)
Sex				
Male	179 (24.0)	341 (45.3)**	71 (9.5)	185 (24.7)**
Female	193 (22.1)	417 (47.8)**	106 (12.1)	215 (24.5)
Age group				
60–64 years	92 (25.8)	177 (48.8)**	42 (11.9)	80 (22.1)**
65–74 years	173 (22.7)	375 (48.2)**	84 (11.1)	203 (25.7)**
≥ 75 years	107 (22.0)*	206 (42.6)**	51 (10.4)	117 (28.5)**
Level of education (%)				
Primary (grade 1–6) or lower	222 (23.0)	19 (48.7)**	100 (10.4)	7 (17.9)**
Middle (grade 7–9) or higher	150 (23.3)	739 (46.6)**	77 (12.0)	393 (24.6)**
All	372 (23.4)	758 (46.8)**	177 (11.0)	400 (24.7)**

* P < 0.05, ** P < 0.01

Table 3 indicates the results of multivariate logistic regression analysis for prevalence of pre-diabetes and diabetes by demographic and lifestyle factors. Current smoker status (OR 1.58, 95% CI 1.11–2.25 ), obesity (OR 1.71, 95% CI 1.27–2.30 ) and central obesity (OR 1.59, 95% CI 1.18–2.15 ) were positively associated with the probability of suffering from diabetes for urban older adults, whereas obesity (OR 1.73, 95% CI 1.30–3.28 ), central obesity (OR 1.83, 95% CI 1.32–2.54 ), and hypertension (OR 2.13, 95% CI 1.54–2.95 ) were positively associated with the prevalence of diabetes for rural older adults. A positive association of obesity with pre-diabetes (OR 2.50, 95% CI 1.53–4.08 ) was only found in the rural participants, while physical inactivity was only found to be positively associated with the prevalence of pre-diabetes (OR 1.95, 95% CI 1.37–2.80 ) in the urban participants.

**Table 3:** Logistic regression of pre-diabetes and diabetes prevalence by demographic and lifestyle factors among rural and urban elderly people of Yunnan Province, China

Variable	Pre-diabetes (reference: no)				Diabetes (reference: no)			
	Rural		Urban		Rural		Urban	
	Odds ratio	95% CI	Odds ratio	95% CI	Odds ratio	95% CI	Odds ratio	95% CI
Sex (reference: male) Age group (reference: 60–64 years)	0.95	(0.70, 1.29)	1.14	(0.89, 1.45)	1.25	(0.83, 1.90)	1.20	(0.90, 1.60)
65–74 years	1.09	(0.88, 1.34)	1.10	(0.88, 1.35)	1.17	(0.90, 1.57)	1.16	(0.89, 1.57)
≥ 75 years	1.05	(0.88, 1.27)	1.07	(0.90, 1.38)	1.12	(0.89, 1.49)	1.09	(0.88, 1.45)
Level of education (reference: primary (grade 1–6) or lower)	0.97	(0.75, 1.25)	0.93	(0.49,1.78)	1.32	(0.94, 1.87)	1.54	(0.67, 3.57)
Current smoker (reference: no)	1.07	(0.75, 1.53)	0.91	(0.67, 1.24)	1.07	(0.64, 1.79)	1.58**	(1.11, 2.25)
Current drinker (reference: no)	1.37	(1.00, 1.87)	1.22	(0.89, 1.67)	0.99	(0.57, 1.73)	1.01	(0.70, 1.45)
Obesity (reference: no)	2.50**	(1.53, 4.08)	1.00	(0.72, 1.40)	1.73**	(1.30, 3.28)	1.71**	(1.27, 2.30)
Central obesity	1.22	(0.93, 1.61)	1.01	(0.77, 1.33)	1.83**	(1.32, 2.54)	1.59**	(1.18, 2.15)
(reference: no)								
Hypertension (reference: no)	1.06	(0.8, 1.36)	0.91	(0.74, 1.11)	2.13**	(1.54, 2.95)	1.12	(0.89, 1.42)
Physical inactivity (reference: no)	1.05	(0.63, 1.72)	1.95**	(1.37, 2.80)	0.81	(0.44, 1.48)	0.78	(0.53, 1.14)

** p < 0.01

## Discussion

This study uncovered significant urban-rural differences in prevalence and determinants of both pre-diabetes and diabetes among older adults in southwest China. Urban older adults experienced markedly higher prevalence of both pre-diabetes and diabetes than the rural elderly, while the associations between pre-diabetes and diabetes prevalence with lifestyle factors varied by region.

The prevalence of pre-diabetes among urban participants (46.8%) was higher than previously measured urban prevalence rates in China (29.5% in Dalian [26] and 18.32% in Guangzhou [27]), as well as in other countries (43.8% in Ecuador [28] and 24.2% in Vietnam [29]), highlighting the scale of the challenge of pre-diabetes in urban southwest China. In contrast, the prevalence of pre-diabetes among rural participants (23.4%) was lower than measured among those of Ningbo in China (30.97%) [30] as well as Daegu in Korea (24.4%) [31]. The prevalence of diabetes in this study was higher than the prevalence of self-reported diabetes (8.7%) in China among both rural and urban older adults [32]. Further, the prevalence of diabetes among urban older adults in this study was also higher than previously (18.8%) [33]. However, some regions of China [12, 27] as well as Myanmar [18] have a higher diabetes prevalence in both urban and rural older adults than measured in the present study. These differences in diabetes prevalence may result from differing regional distribution of dietary patterns, economic levels, living environments, age groups, and ethnicity [34], as well as differing definitions of diabetes. Our findings indicate that a large population of urban southwest Chinese residents faces a high risk of progression of their pre-diabetes to diabetes, underscoring the need for interventions, such as lifestyle modifications and treatments, to prevent the development of diabetes among pre-diabetic older adults.

In the present study, prevalence of pre-diabetes and diabetes in the urban older adult population was much higher than the rural population, consistent with findings from previous studies in China [6, 7] as well as studies conducted in other countries [18, 19]. This may result from the fact that urban older adults had a significantly higher prevalence of obesity and central obesity than their rural counterparts (15.3% and 76.0%, vs. 4.6% and 45.6%), and that elderly people living in urban China tended to be less physically active during the day than rural elder adults (9.2% vs. 6.1%). The findings in this way highlight an urgent need to address urban lifestyle habits that create the conditions for obesity and sedentariness as well as the importance of surveillance of urban diabetes patterns in order to head off an emerging diabetes epidemic in urban southwest China.

The present study also found that obesity and central obesity were positively associated with the probability of suffering from diabetes for both urban and rural older adults. The significant role of obesity and central obesity in contributing to the development of diabetes is well established in the literature [7, 15, 35]. However, the association of obesity with pre-diabetes in the present study was only found in the rural area. Further research is needed to uncover the roots of this inconsistent effect of obesity on pre-diabetes development between urban and rural areas.

Our study additionally uncovered that while the prevalence of current smoking was higher in the rural region, current smoking status was positively associated with the probability of having diabetes among urban older adults but not among rural ones. This finding of an inconsistent effect of smoking on diabetes aligns with previous research [14, 18, 36, 37]: most studies have reported a positive association between smoking and diabetes, but some researchers have found a lower prevalence of diabetes among smokers than nonsmokers. The reasons behind this dichotomy are unclear and require further research.

Among the urban elderly participants in this study, those lacking in daily physical activity were more likely to suffer from diabetes. It is well known that inadequate physical activity as well as irregular living habits contribute to the prevalence of obesity, which may lead to the development of metabolic diseases, including diabetes [38]. There is also robust evidence of the beneficial effects of physical exercise on preventing metabolic disease and cardiovascular disease [39]. Thus, regular physical exercise should be promoted to reduce the prevalence of diabetes among elderly residents of urban southwest China.

Our study results showed that while the prevalence of hypertension was not significantly different between rural and urban older adults, the rate of hypertension, as diagnosed by a healthcare professional, among the urban elderly was obviously higher than the rural elderly, contributing to rural-urban health disparities in southwest China [40]. Moreover, rural hypertensive older adults were more likely to suffer from diabetes than urban ones. This may result from the fact that hypertensive patients in rural areas have developed unhealthy eating habits (with diets high in fat and sodium) [41], as hypertension is an independent risk factor for diabetes [42]. The study findings indicate that increasing access to healthcare and improving health literacy among the rural elderly population remains an important strategy to improve health outcomes in rural China.

There are several limitations to the present study. First, the data analyzed were from a cross-sectional study, so causal relationships cannot be determined. Second, the present study might underestimate the true prevalence of pre-diabetes and diabetes as only FBG was used to diagnose diabetes and pre-diabetes, and hemoglobin A1C and postprandial blood glucose were not measured. Third, variables such as sleep quality and depressive disorder, considered potential risk factors for diabetes, were not measured in this study. Fourth, as the present study did not consider information regarding the proportion of diabetic patients who were migrants to include, the true rural-urban differentials of prevalence of diabetes might be biased. Finally, our research enrolled the elderly population in one district and one county of Yunnan Province, which may not fully represent the entire elderly population of southwest China. More data are needed to expand our findings.

In conclusion, our study indicated that pre-diabetes and diabetes are more prevalent among urban older adults than rural older adults in southwest China. Further, our results uncovered rural-urban differentials of lifestyle factors that have significant impacts on prevalence of pre-diabetes and diabetes. Tailored lifestyle interventions are needed to improve diabetes prevention and management in southwest China into the future.

## Data Availability

The datasets used and/or analyzed in the present study are available from the corresponding author on request.
